# Large gaze shift generation while standing: the role of the vestibular system

**DOI:** 10.1152/jn.00343.2019

**Published:** 2019-09-04

**Authors:** Dimitri Anastasopoulos, Nausika Ziavra, Adolfo M. Bronstein

**Affiliations:** ^1^Department of Neurology, University of Ioannina, Ioannina, Greece; ^2^Akutnahe Rehabilitation, Kantonsspital Baden, Baden, Switzerland; ^3^Department of Speech and Language Therapy, University of Ioannina, Ioannina, Greece; ^4^Department of Brain Sciences (Neuro-otology Unit), Imperial College London, Charing Cross Hospital, London, United Kingdom

**Keywords:** anticompensatory, bilateral vestibular loss, coordination, gaze, multisegmental, turns

## Abstract

The functional significance of vestibular information for the generation of gaze shifts is controversial and less well established than the vestibular contribution to gaze stability. In this study, we asked seven bilaterally avestibular patients to execute voluntary, whole body pivot turns to visual targets up to 180° while standing. In these conditions, not only are the demands imposed on gaze transfer mechanisms more challenging, but also neck proprioceptive input represents an inadequate source of head-in-space motion information. Patients’ body segment was slower and jerky. In the absence of visual feedback, gaze advanced in small steps, closely resembling normal multiple-step gaze-shift patterns, but as a consequence of the slow head motion, target acquisition was delayed. In ~25% of trials, however, patients moved faster but the velocity of prematurely emerging slow-phase compensatory eye movements remained lower than head-in-space velocity due to vestibuloocular failure. During these trials, therefore, gaze advanced toward the target without interruption but, again, taking longer than when normal controls use single-step gaze transfers. That is, even when patients attempted faster gaze shifts, exposing themselves to gaze instability, they acquired distant targets significantly later than controls. Thus, while patients are upright, loss of vestibular information disrupts not only gaze stability but also gaze transfers. The slow and ataxic head and trunk movements introduce significant foveation delays. These deficits explain patients’ symptoms during upright activities and show, for the first time, the clinical significance of losing the so-called “anticompensatory” (gaze shifting) function of the vestibuloocular reflex.

**NEW & NOTEWORTHY** Previous studies in sitting avestibular patients concluded that gaze transfers are not substantially compromised. Still, clinicians know that patients are impeded (e.g., looking side to side before crossing a road). We show that during large gaze transfers while standing, vestibularly derived head velocity signals are critical for the mechanisms governing reorientation to distant targets and multisegmental coordination. Our findings go beyond the traditional role of the vestibular system in gaze stability, extending it to gaze transfers, as well.

## INTRODUCTION

In all mammals, stability of the visual scene during head movements is guaranteed by vestibular information. Motion-provoked oscillopsia (oscillation of the visual world) and visual blur are regularly reported by patients with bilateral vestibular loss (BVL), symptoms not only substantially compromising physical functioning and quality of life (Grunfeld et al. 2000) but also linked to an increased risk of falls (Schlick et al. 2016; Schniepp et al. 2017).

Gaze (eye-in-space) saccades after sudden loss of vestibular function in primates are accompanied by a dramatic impairment of both target acquisition, (i.e., overshoot) and postsaccadic stabilization of the line of sight (Dichgans et al. 1973). Humans with chronic vestibular deficits, however, adapt to the vestibuloocular reflex (VOR) loss by developing compensatory mechanisms (Kasai and Zee 1978). Visuomotor performance in such patients has been found to be only marginally compromised; the temporal coupling of eye and head movements is similar to that of normal subjects, and patients are thought to regain the ability to make functionally appropriate gaze saccades (Maurer et al. 1998). Only when the head inertia is unexpectedly increased (for example, by artificially adding weight) do patients display large head and gaze oscillations (Lehnen et al. 2009). Critically, however, all these primate and human studies have been performed in sitting conditions, a great paradox given that vestibular input is chiefly needed when one is up and about.

Much is known about vestibular input to gaze stability during head movements (compensatory function of the VOR). However, vestibular effects during the execution of gaze shifts and their contribution to gaze accuracy are controversial; this is the so-called “anticompensatory” function of the VOR, which is brought about by the fast phase components of vestibular nystagmus into the direction of head displacement (Mishkin and Jones 1966). Gaze feedback hypotheses suggest that a vestibularly derived signal monitors head motion such that VOR reactivation (and gaze stabilization on the target) can occur when gaze is near the target (Boulanger et al. 2012; Daye et al. 2014; Guitton and Volle 1987; Laurutis and Robinson 1986). Alternatively, hypotheses relying more on feedforward control propose that gaze amplitude is not controlled directly. According to these views there is no need for sensory input from vestibular sensors to generate appropriate gaze shift size and secure arrival of the visual axis on the target (review by Freedman 2008).

Previous measurements in patients with chronic bilateral vestibular loss may have failed to establish whether vestibular mechanisms shape online gaze displacements because, as mentioned, investigations were made only while patients were sitting. In contrast, in the present study we have included movements of the trunk and feet during voluntary pivot turns to both visual and predictable targets at large eccentricities up to 180°. This is a most ecological condition (Land 2004) but also one in which neck proprioceptive signals represent an inadequate source of head-in-space motion information (because the trunk is moving as well) and stronger demands are imposed on the control mechanism (because of the greater eccentricities and large body segments’ inertia). For these reasons, previous studies may have failed to detect gaze shift defects in avestibular patients, thus falling short of confirming if the vestibular system has a role in target acquisition.

In healthy subjects, large-eccentricity target acquisitions during standing can be accomplished as accurate single-step shifts of the visual axis (note that single step denotes gaze, not foot steps) covering at least 85% of the required visual angle (see Fig. 2*B*; Anastasopoulos et al. 2009). Head and trunk displacements are coupled at a kinematic level (Sklavos et al. 2010). More frequently, however, normal subjects generate less time-efficient multiple-step gaze transfers, whereby one or more short intervals of stationary gaze emerge before target acquisition. We hypothesized that under these experimental conditions, derangements of the normal gaze trajectory pattern would appear if head velocity information were important for gaze control. Also, by dissociating head-on-trunk from head-in-space motion, we expected that the contribution of the neck proprioceptive input to adaptive compensatory mechanisms during an ecological task could be directly exposed. In sum, we expected to be able to examine for the first time how bilateral vestibular failure impacts on both the compensatory and, in particular, the anticompensatory functions of the VOR during a natural, upright gaze-transfer task.

## MATERIALS AND METHODS

### Patients and Control Subjects

Measurements in seven patients (6 men, age 52 ± 11 yr, mean ± SD) with long-standing bilateral vestibular loss were compared with those in 10 control subjects previously reported (52 ± 2.6 yr; 7 men; Anastasopoulos et al. 2009). Patients had suffered bilateral vestibular loss (5 idiopathic, 1 due to neurosarcoidosis, and 1 following gentamicin intoxication) at least 7 yr before measurements. Vestibular areflexia was documented by caloric irrigation with water at 30°C and 44°C, 120°/s velocity steps on a motorized Bárány chair, and a clinically positive three-dimensional head impulse test. One patient’s responses were reduced to <10% of the normal level, whereas all the rest did not respond even on irrigation at 20°C. No subject wore spectacles, and selection was careful to guarantee good physical condition. Control subjects were free of visual, vestibular, or neurological disease. All subjects were right-hand/foot dominant (Waterloo Footedness Questionnaire-Revised; Elias et al. 1998). All subjects provided informed consent according to the Declaration of Helsinki as approved by the IC Riverside Ethics Committee.

### Experimental Design and Data Acquisition

**Fig. 1.**
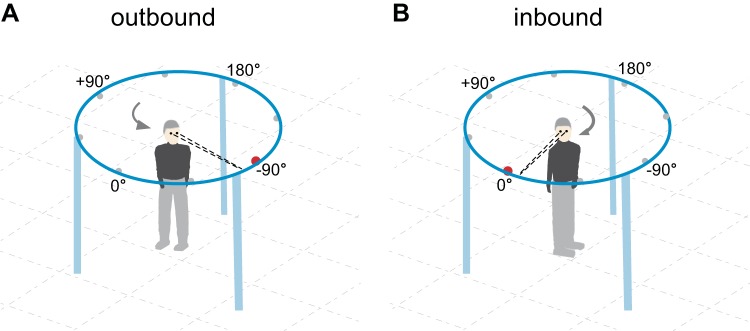
Experimental setup. At the beginning of the trial, the subject had aligned himself with the central light-emitting diode (LED) at 0°. After a delay of 10 s, an eccentric LED (shown at −90°) was lit while the central LED was turned off, and the subject started to turn to align his whole body with it (outbound trial; *A*). Note that the lower extremities started to move later than any other segment and are shown in the schematic to be still directed toward the central target at 0°. After the subject had assumed the new orientation in space, the eccentric LED was turned off while the central LED was turned on, thus cueing the subject to return to the initial, now predictable direction in space (inbound or “return” trial; *B*). The in-house-made octagonal wooden frame, which was installed within the LED array to prevent subjects from falling, is not depicted in the schematic.

Participants stood in the center of a circular array (radius 1.2 m) of 8 light-emitting diodes (LEDs), placed at 45° intervals at eye level, in dim lighting (Fig. 1). An in-house-made octagonal wooden frame (handrail 93 cm from the ground, 93 cm between each of the parallel sides) was installed within the LED array to prevent subjects from falling and to help subjects feel secure when turning. No patient fell, “near” fell, or appeared severely unstable during measurements. At the beginning of each trial, subjects were required to fixate and align their head, body, and feet with the central LED. After a delay of 10 s, the central target was extinguished, thus indicating that an eccentric LED in one of seven locations (±45°, ±90°, ±135°, and 180°) had been lit. At this point, subjects had to align their whole body with the new target (outbound trials). After an interval of 10 s, the eccentric LED was turned off, thus cueing subjects to return to the initial, centrally positioned LED (inbound or “return” trials are thus spatially predictable). This protocol guaranteed that, due to the obligatory trunk movement, neck proprioceptive information from head-on-trunk displacement did not indicate head-in-space motion, as was the case in previous work with sitting BVL patients (e.g., Bronstein and Hood 1986). Also, by assuming a defined starting position of the eyes near primary gaze before a trial, variability in initial orbital eye position was not a confound. Note that trials to 45° targets are visually driven, but for 90° or larger trials, the target is initially not visible and patients had no hint as to its location during outbound trials or in which direction they should turn. Consequently, in such cases subjects often turned first in the wrong direction (~50%) and then, after realizing their mistake, moved abruptly back in the opposite direction. Data from such “wrong-direction” trials were not further analyzed because of the variable relationship between the eye/head/trunk initial positions. During inbound (recentering, return) trials, however, subjects knew where the centrally positioned target was to be found. Subjects performed in random order four trials to each LED location. Turns were accomplished at natural, freely chosen speeds rather than subjects being forced to execute time-optimal movements. Our previous study showed normal intra- and intersubject strategy variability (Anastasopoulos et al. 2009), and we wanted to see whether the strategy of patients with loss of vestibular function would differ from that of healthy subjects.

Head-in-space, upper trunk, and feet horizontal angular movements were recorded using a Polhemus Fastrak motion analysis system. The trunk marker was also set to record forward-backward and left-right linear movements in a space-fixed coordinate system. Horizontal eye-in-head rotations were recorded using bi-temporal direct current electrooculography (EOG; flat response between 0 and 90 Hz). Initial dedicated eye-in-orbit calibrations were obtained by asking subjects to fixate targets at ±10–30°. They were also often subsequently asked to turn their heads horizontally through various angular displacements up to 45° while fixating the central target. EOG was then related to the signal obtained from the head marker. On-off target signals, EOG, and body sensors were sampled at 240 Hz and stored for off-line analysis. Eye position (EOG) and head position (Fastrak) signals were added to obtain “gaze,” namely, an approximation of absolute eye position in space. In turn, subtracting trunk from head signals yields a head-on-trunk movement signal. A smoothing Savitzky–Golay filter was used for off-line velocity measurements such that significant portions of the signal’s high-frequency contents (up to 25 Hz) were not filtered out. End-point jerk cost for body segment motion was calculated as

$$JC={\int_{0}^{T}\left(\frac{d^{3}r}{dt^{3}}\right)}^{2}dt,$$

where *T* is the time interval from movement onset until movement end and *r* is the segment angular position vector in the horizontal plane. Jerk (in deg/s^3^) was calculated from unfiltered signals with MATLAB (The MathWorks). Further experimental details are given in previous reports (Anastasopoulos et al. 2009).

### Statistical Analysis

Because multiple correlated measurements were made for each subject, a linear mixed effects analysis was performed to outline the relationship between the dependent variable of interest **Y** (i.e., movement latency/amplitude/velocity, end point jerk cost, target acquisition time) and patients vs. controls (group). Random intercept models were used with intercepts for subjects being entered as random effects (*u*). Target eccentricity (45°, 90°, 135°, 180°), target presentation [comparison of responses to *1*) visible visual targets of 45°, *2*) initially nonvisible 90°, 135°, and 180° outbound targets, and *3*) initially nonvisible 90°, 135°, and 180° return targets], body segment (eye, head, trunk, foot), and group (patient, control) were introduced into the models as fixed effects (**β**) with or without interaction terms.

In matrix notation,

Y=Xβ+Zu+ε,

where **Y** is the *n* × 1 vector of responses, **X** is an *n* × *p* design/covariate matrix relating the responses to the fixed effects **β**, and **Ζ** is the *n* × *q* design/covariate matrix relating the responses to the random effects *u* (Stata SE 14.1; StataCorp). Chi-square values were rescaled to *F* ratios because of the small sample size. Additional proof of significance was derived by likelihood ratio tests comparing the full model with the effect of a fixed term (i.e., group) against the reduced model without the effect of the same term. A significant difference between the likelihood of these two models would confirm that the fixed term under consideration affects the dependent variable. Deviations from homoscedasticity were estimated by visual inspection of residual vs. fitted value plots. Data are generally reported as medians and interquartile ranges (Q_25_–Q_75_). Neither patient nor control data were asymmetrical; thus responses to targets to −90° and −135° were pooled with those to +90° and +135°, respectively.

## RESULTS

### Response Patterns

**Fig. 2.**
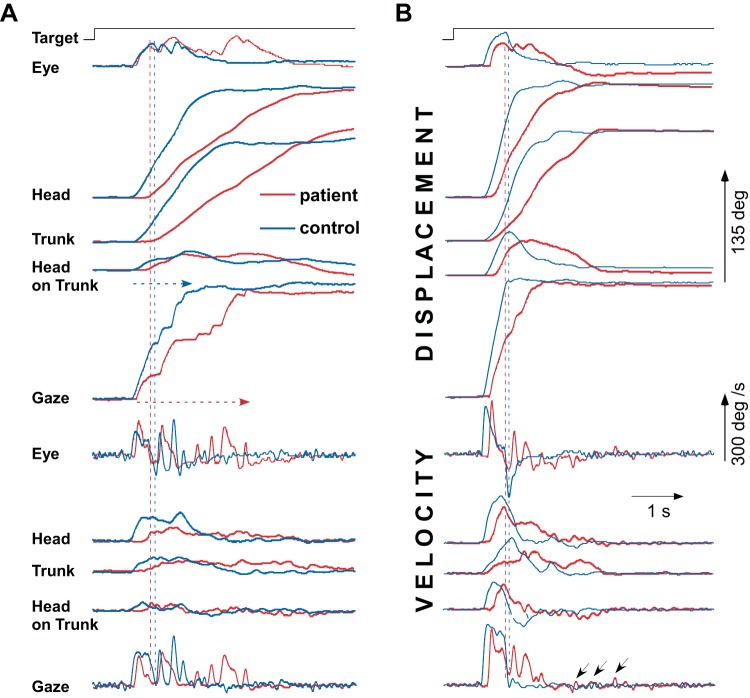
Representative examples of rightward inbound multiple-step (*A*) and single-step (*B*) gaze shifts to the central target at 135° eccentricity by a patient (red traces) and a control subject (blue traces). Position and velocity traces are shown at *top* and *bottom*, respectively. Vertical dashed lines indicate the termination of the primary gaze shift by a leftward (downward) slow eye movement. Note the shorter head-on-trunk and trunk contribution to gaze displacement in the patient compared with those in the control subject. *A*: gaze continued to shift toward the target by the sum of fast phases and head-in-space displacement. Horizontal dashed arrows indicate acquisition time (considerably longer in the patient). Slow-phase eye velocity in the patient is approximately equal and opposite to head-in-space velocity. *B*: the velocity of the first premature and a subsequent slow-phase eye movement in the patient is considerably lower than head-in-space velocity so that gaze continues to advance toward the target. In the control, the emergence of the slow phase indicates the termination of gaze displacement and target acquisition. Oblique arrows (*bottom* trace) indicate corrective saccades for the gaze drift after target acquisition in the patient.

Details of movement patterns in normal subjects have been described in previous reports (Anastasopoulos et al. 2009, 2015; Sklavos et al. 2010). As a general summary, movement initiation, metrics, and kinematics depend on target eccentricity and predictability. Visual targets at 45° eccentricity, which are fully visible, are usually foveated by combined eye and head-on-trunk displacements, whereas the contribution of trunk rotation is negligible. Trunk contribution to gaze displacement increases when more eccentric and particularly predictable targets are attained. In spatially predictable conditions (i.e., inbound 90°, 135°, and 180° targets), normal subjects frequently execute single-step gaze shifts (Fig. 2*B*). Even under these conditions, however, they mostly acquire distant targets with the “scanning” multiple-step pattern (i.e., in 40, 65, and 75% of these trials, respectively; Fig. 2*A*). This pattern is the only one used under spatially unpredictable conditions (i.e., outbound 90°, 135°, and 180° targets), when visual scanning is essential to locate the target.

In patients, as a rule, body segment movements started to move later, and both peak head-in-space and trunk velocities were lower than in controls. Table 1 summarizes all variables measured in both subject groups and the relevant statistical comparisons. Visual targets at 45° were attained mostly with a large eye saccade. Patients used, just as controls, predominantly the multiple-step scanning pattern when trying to attain both predictable and guessed targets. Less frequently (40, 20, and 10% of trials to 90°, 135°, and 180° predictable targets, respectively), gaze displacement consisted not only of saccadic but also of “slow-phase” eye rotation components, directed opposite to head motion. During these slow-phase segments, eye velocity was, however, considerably lower than head-in-space velocity (due to the absence of VOR) such that their sum still resulted in net gaze displacement toward the target, without pauses, i.e., without periods of gaze stability (“non-pause” composite gaze shifts). The initial (primary) gaze saccade to predictable targets was significantly shorter in patients, and as a consequence of all these changes, they acquired the target significantly later than controls (see Fig. 4).

**Table 1.** Movement parameters and comparisons between BVL patients and normal controls

	BVL Patients	Controls	Test	Significance
Trunk sway path before movement initiation, mm/s	5.5 [4.0–6.5]	4.7 [3.4–5.5]	lme; lr	ns
Trunk sway path after visual target acquisition, mm/s	17.5 [15.5–21.7]	8.6 [7.8–9.8]	lme; lr	*F*_(1,15)_ = 21.99, *P* = 0.0003; χ^2^ = 13.6, *P* = 0.0002
Trunk sway path after predictive target acquisition, mm/s	31.2 [26.2–38.5]	17.6 [15.9–18.7]	lme; lr	*F*_(1,15)_ = 24.22, *P* = 0.0002; χ^2^ = 14.6, *P* = 0.0001
Latency of movement as a whole, s			lme; lr	*F*_(1,15)_ = 6.64, *P* = 0.02; χ^2^ = 5.6, *P* = 0.018
Latency, s: of target presentation × group			lme; lr	*F*_(2,15)_ = 8.2, *P* = 0.004; χ^2^ = 15.7, *P* = 0.0004
Eye saccade amplitude, deg	30.7 [22.0–35.5]	30.3 [26.6–36.0]	lme	ns
*Visual targets at 45° eccentricity*				
Latency, s				
Eye	0.39 [0.28–0.71]	0.42 [0.36–0.53]	lme; lr	ns
Head-on-trunk	0.51 [0.44–0.79]	0.52 [0.45–0.63]		
Trunk	0.67 [0.54–1.06]	0.63 [0.59–0.76]		
Earliest foot	1.19 [1.01–1.58]	0.99 [0.87–1.10]		
Initial gaze shift amplitude, deg	37.2 [33.1–37.8]	37.9 [34.8–41.6]	mw	ns
Peak head-in-space velocity, deg/s	67 [52–105]	109 [99–126]	mw	*P* = 0.04
Peak trunk velocity, deg/s	50 [47–54]	65 [58–86]	mw	*P* = 0.008
Acquisition time, ms	430 [380–482]	390 [332–430]	mw	ns
*Predictable targets at 90°, 135°, and 180° eccentricity*				
Latency, s				
Eye	0.62 [0.54–1.34]	0.51 [0.46–0.61]	lme; lr	*F*_(1,15)_ = 5.45, *P* = 0.03; χ^2^ = 4.7, *P* = 0.03
Head-on-trunk	0.75 [0.61–1.92]	0.62 [0.48–0.72]		
Trunk	0.73 [0.65–1.22]	0.68 [0.52–0.75]		
Earliest foot	1.14 [1.08–1.44]	0.88 [0.68–1.08]		
Initial gaze shift amplitude, deg	37.9 [17.1–49.8]	74.3 [52.9–99.7]	lme; lr	*F*_(1,15)_ = 7.43, *P* = 0.016; χ^2^ = 6.11, *P* = 0.013
Peak head-in-space velocity, deg/s	120 [96–168]	199 [159–234]	lme; lr	*F*_(1,15)_ = 5.91, *P* = 0.03; χ^2^ = 5.10, *P* = 0.02
Peak trunk velocity, deg/s	95 [76–103]	123 [110–147]	lme; lr	*F*_(3,15)_ = 10.85, *P* = 0.005; χ^2^ = 8.16, *P* = 0.004
Head end-point jerk cost, deg^2^/s^5^	4.66 [3.79–5.87] × 10^7^	3.39 [2.10–4.41] × 10^7^	lme; lr	*F*_(1,15)_ = 4.67, *P* = 0.05; χ^2^ = 4.2, *P* = 0.04
Trunk end-point jerk cost, deg^2^/s^5^	3.69 [2.24–6.02] × 10^7^	2.05 [1.33–3.00] × 10^7^	lme; lr	*F*^(1,15)^ = 16.67, *P* = 0.001; χ^2^ = 11.7, *P* = 0.0006
Acquisition time, ms	1,605 [980–2,083]	935 [760–1,308]	lme; lr	*F*_(1,15)_ = 8.79, *P* = 0.001; χ^2^ = 7.08, *P* = 0.008

Values are medians and interquartile ranges. BVL, bilateral vestibular loss; lme, Linear mixed effects (*F* ratio); lr, likelihood ratio test (χ^2^); mw, Mann–Whitney *U* test; ns, nonsignificant; ×, interaction between two variables..

Figure 2*A* exemplifies the most common movement pattern in patients and the striking similarities with those of the controls, whereas Fig. 2*B* captures the main differences in the non-pause patterns of combined movement between the groups. Importantly, five patients, including the patient with residual vestibular function, foveated predictable targets by either non-pause (less frequently) or multiple-step gaze transfers, whereas the remaining two patients executed exclusively multiple-step patterns.

### Movement Initiation (Latencies)

As a whole, patients began to move significantly later than controls as estimated by a linear mixed effects model with target presentation (visual, outbound, return), body segment (eye, head, trunk, foot), and group (BVL, normal) as fixed effects and intercepts for subjects as random effects (Table 1). The interaction between the factors target presentation and group was significant, i.e., latencies to visual targets (45°) in patients were normal, whereas those to nonvisible targets (>45°) were prolonged (Table 1; up to ~100–150 ms in return trials).

### Metrics of Rotational Movement

#### Eye saccades, gaze displacement, and head-on-trunk deflections.

The amplitude of the initial (primary) eye saccade was normal in patients (Table 1). Also, primary eye saccade velocity (for eye saccade amplitude 25–30°, i.e., in the range when peak velocity becomes largely independent of the amplitude) was not different in the two groups, as expected.

**Fig. 3.**
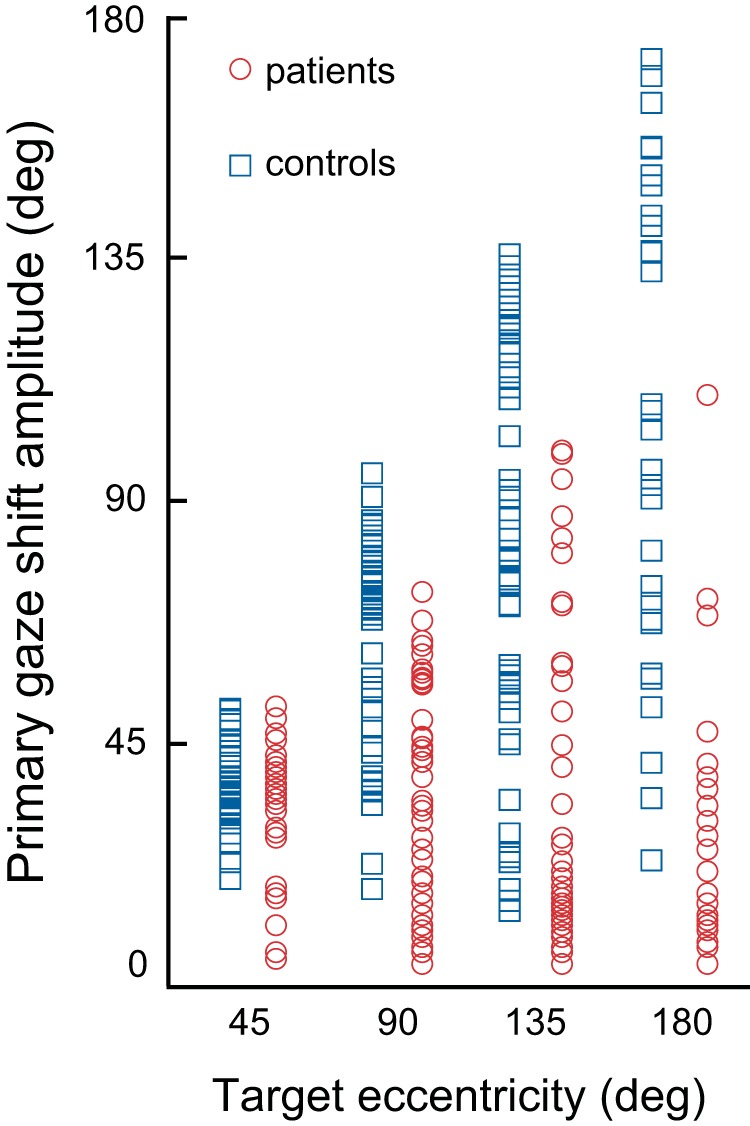
Initial (primary) gaze shift amplitude. Distribution of data from all individual return trials in patients (red circles) and controls (blue squares) when target location was either visible (45**°**) or predictable (90°, 135°, and 180°). Note that in patients, the saccadic gaze transfer always terminated far before covering the target eccentricity.

Whereas the amplitude of the initial gaze shift to visual targets (45°) approached 40° and was normal in patients, gaze saccades to 90°, 135°, and 180° predictable (return) targets were significantly shorter (Table 1; Fig. 2, dashed vertical lines; Fig. 3). Gaze velocity saturated at gaze amplitudes >60° and was not significantly different between the patient and control groups.

There was no statistically significant difference of head-on-trunk movement amplitude between the groups, amounting to ~45° and 30° in outbound and inbound trials to nonvisible targets, respectively.

#### Head-in-space and trunk velocity.

Peak head-in-space velocity was generally lower in patients, and the difference from controls reached statistical significance in trials to both visual and predictable targets (Table 1). Peak trunk velocity was similarly reduced in patients both in trials to visual targets at 45° and in predictable trials to 90°, 135°, and 180° targets. Because sway path in the horizontal plane, as measured from the translational displacement of the trunk marker, was normal in patients during the interval of 10 s before movement initiation, i.e., during fixation of the lit LED (Table 1), it is unlikely that these differences are related to hesitancy derived from instability.

Notably, the head and, most particularly, trunk velocity profile in patients was less smooth than that in controls, characterized by abrupt fluctuations after peak velocity was achieved (compare exemplary traces in Fig. 2). Non-smooth movements require abrupt changes of muscle force leading to increased control-dependent noise and, in consequence, increased end-point variability of the moving segments. Because the central LED remained lit during return trials, movement end-point variability was not a useful parameter to decide whether the lack of online head velocity feedback in patients may have resulted in larger end-point variability. We therefore quantified head and trunk trajectory smoothness by evaluating the jerk cost function (Flash and Hogan 1985) over the first four consecutive return trials. End-point jerk cost increased significantly with increasing target eccentricity in both groups. Patients exhibited normal values when reorienting to visual targets at 45°, but significantly higher values when returning to 90°, 135°, and 180° predictable targets (Table 1). Still, sway path after rotations to visual and predictive targets was significantly increased in patients, suggesting that voluntary reorientations even to short eccentricities and under visual feedback may impose the risk of losing balance (Table 1).

#### Contribution of head and trunk to the initial gaze shift and the emergence of slow and fast phases.

Visual axis reorientations to visible targets at 45° could be accomplished by the sum of a large saccade and a small head-on-trunk displacement. Thereafter, head, trunk, and lower extremities completed the reorientation task at a slower pace. The situation was quite different when subjects had to move to distant eccentricities beyond the oculomotor range (initially nonvisible, either predictable or outbound targets) because they were forced to rotate head and trunk before target acquisition. In most of these cases, both patients and controls used the scanning, multiple-step pattern of gaze displacement, whereby the initial eye saccade was soon terminated by an oppositely directed slow eye rotation. Thus the initial gaze shift would cover only a small fraction of the angular distance to the predicted target location (Fig. 2*A*, vertical dashed lines). In controls, gaze continued thereafter to shift toward the target by the sum of head-in-space displacement and repetitive fast eye movements. These were interrupted by oppositely directed slow phases of vestibular nystagmus such that gaze was thereby stationary (Anastasopoulos et al. 2009). Slow-phase eye velocity in patients was also roughly matched to head-in-space velocity so that gaze was occasionally stabilized in space, particularly when head-in-space velocity was low (compare *bottom* trace in Fig. 2*A*). Importantly, it did not correlate with head-on-trunk velocity. Gaze was forwarded toward the target again by the sum of a number of fast phases with head and trunk rotation. In many such cases, the intersaccadic (or internystagmic) interval was often <100 ms, suggesting they are not truly “voluntary.”

Patients and control subjects could acquire distant predictable targets relatively rapidly and without intervals of stabilized gaze in space by increasing head-in-space velocity (Fig. 2*B*). The controls would cover in such cases more than 85% of the displacement to the target with a single large saccade, terminated by a slow-phase eye movement only when gaze was very close to the target (and thus stabilizing gaze on the target despite the continuing head motion, single-step pattern; Anastasopoulos et al. 2009; Fig. 2*B*, blue traces). The non-pause patterns of combined multisegmental movement in patients are only partly comparable to the single-step patterns in controls because they comprise fast and oppositely directed slow phases, the earliest slow phase emerging prematurely, i.e., in midflight. However, because throughout these occasions slow-phase eye velocity was considerably less than head-in-space velocity (i.e., incompletely compensatory for head velocity), gaze advanced further without stabilization intervals (Fig. 2*B*, red traces). Thus, in patients, both when using the low head-in-space velocity multiple-step or the high-velocity non-pause pattern, the initial (primary) gaze shift consisted mainly of displacement of the eye, whereas head-on-trunk and trunk contributed little to it, resulting in significantly shorter gaze shifts (Fig. 3).

### Task Performance

**Fig. 4.**
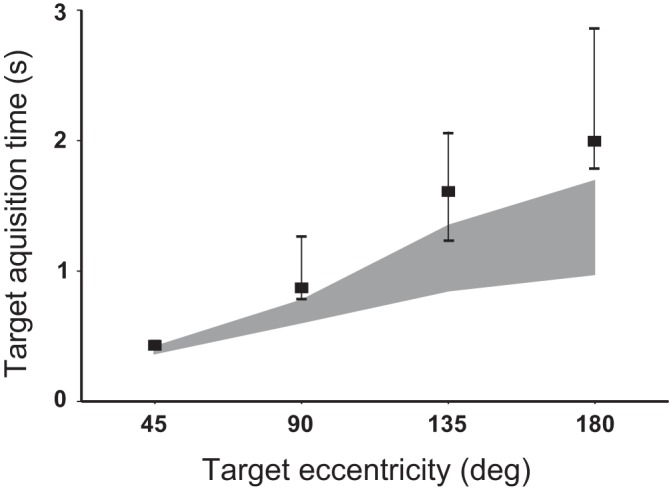
Prolonged acquisition time of 90°, 135°, and 180° targets in the patient group. Shaded areas represent interquartile range of normal values, whereas values for patients with bilateral vestibular loss are shown by quadrangles and error bars (median and interquartile ranges). Visual targets at 45° were acquired by patients as quickly as by normal subjects.

Performance was defined as target acquisition time when the target could be visually perceived or predicted, i.e., reorientations to guessed targets were excluded (horizontal arrows in Fig. 2). Patients’ performance was considerably impaired (Table 1 and Fig. 4), with patients needing on average 140, 250, and 700 ms more than controls to acquire 90°, 135°, and 180° targets, respectively. In contrast, visual targets (45°) were acquired as quickly as in normal subjects. Finally, postsaccadic gaze stability was characterized by a number of corrective secondary saccades during the 1.5-s time interval immediately after gaze velocity had crossed zero. Patients exhibited slow gaze drifts, most particularly after fast non-pause gaze shifts to predicted targets, which were corrected by means of two to four small secondary saccades (controls produced just one corrective saccade at most; Fig. 2*B*, *bottom* trace, small arrows).

## DISCUSSION

Eye-head coordination in bilateral vestibular loss patients has been previously examined only in sitting subjects (Bronstein and Hood 1986; Kasai and Zee 1978; Maurer et al. 1998; Sağlam et al. 2014). In the present study, we asked patients to re-foveate targets at large eccentricities while standing upright, i.e., an experimental paradigm more likely to reveal the functional significance of the loss of head velocity information for gaze transfers and, at the same time, more ecological and clinically meaningful.

Trunk and head-in-space movements were, as a rule, irregular and slower in patients. During large-eccentricity outbound and inbound target jumps (when targets are not visible), movement latencies were prolonged, body movements were more “noisy,” and coordination of the eye and body segments was less efficient, with the consequence of significant foveation delays. Compensation for the vestibular failure was achieved by using the slower multiple-step gaze-transfer strategy through the generation of fast and slow eye movements. Faster target acquisition was compromised by patients’ inability to timely generate slow phases matching higher head-in-space velocity. Faster displacements may be therefore presumably possible only at the cost of increased retinal image slippage and, therefore, oscillopsia. Notably, all patients reported oscillopsia during such head movements in daily life.

### BVL Patient Strategies for Target Acquisition

Patients covered the distance to visual targets at 45° easily, mainly by means of a large eye saccade (Maurer et al. 1998). The normal segmental latencies when patients foveated visible visual targets (45°), in contrast to the significantly prolonged reaction times to nonvisible targets ( ≥90°), can be attributed at least partly to the fact that the former were supported by visual feedback. Patients preferred to move the head and trunk mostly after target acquisition, just as normal subjects did. Gaze stabilization during the postacquisition time interval was on these occasions good, and this was certainly assisted not only by the low head-in-space velocity but also through the activation of additional sources of slow-phase eye movements such as the cervicoocular reflex (COR) and smooth pursuit as the target remained lit (Bronstein and Hood 1986; Dichgans et al. 1973).

The rapid acquisition of initially nonvisible targets at 90°, 135°, and 180° requires, however, early body segmental mobilization, as shown in Fig. 2 and our previous studies (Anastasopoulos et al. 2009). Preparing the coordination of a large number of effectors requires in this case more processing time, even in normal subjects (Scotto Di Cesare et al. 2013). In BVL patients, this process is likely to be further complicated by the lack of online vestibular input controlling the multisegmental motion; simply put, the head is moving, but the brain cannot tell exactly how fast.

In most trials patients used the multiple-step, visual scanning gaze pattern for both outbound and predicted (return) targets. The close resemblance of many aspects of patients’ performance to that of control subjects was remarkable (Fig. 2*A*). Probably as an adaptive strategy, both head-in-space and trunk velocities were, however, held conspicuously low, with the result that target acquisition was considerably delayed; this is a handicap never previously reported or indeed suspected in these patients. The displacement of the line of sight toward the target was in these instances interrupted by intervals of eye rotation opposite to head movement. These vestibularly derived intervals in normal subjects mediate gaze stability during head motion and are suitable for visually scanning the environment during roaming head movements (Anastasopoulos et al. 2009). The velocity of such slow phases in patients matched approximately, although not perfectly, head-in-space velocity but did not change much throughout the movement, suggesting that they are possibly generated by predictive mechanisms. It is unlikely that slow phases in patients are based solely on head-on-trunk proprioceptive information (i.e., the COR), because they did not correlate with head-on-trunk motion (Fig. 2). Indeed, the COR cannot be the exclusive source of compensatory eye movements when, just as in this task, head-in-space does not coincide with head-on-trunk velocity. In contrast, the often short intersaccadic interval of fast phases, alternating at short intervals with the slow phases, argues against them being voluntarily triggered. They therefore are likely to be generated by reflex mechanisms, for instance, by the so-called anticompensatory function of the COR. Fast phases in the direction of head turn, generated at short latency in response to unpredictable head turns in patients devoid of vestibular function, has earlier been taken as evidence that in such patients neck input (COR) takes over the anticompensatory function of the VOR (Bronstein and Hood 1986).

Less frequently, patients would increase head and trunk velocity and shorten significantly the acquisition of the target by combining fast- and slow-phase intervals in the same direction of gaze transfer. Because in these non-pause composite gaze transfers (e.g., “non-pause” because gaze velocity toward the target never comes to a standstill) the velocity of oppositely directed slow eye rotations was usually only a fraction of the concurrent head-in-space velocity, considerable retinal slip and oscillopsia would occur. This is what patients report, for instance, when crossing a road and looking quickly from side to side (Grunfeld et al. 2000). Moreover, due to the increased head-in-space velocity, undisturbed fixation of the target during the postsaccadic time interval was frequently disrupted, as supported by the increased number of secondary corrective saccades in these cases (Fig. 2*B*). Patients may therefore have to trade-off (“decide”) between delayed target foveation in a frame of acceptable space constancy or shorter acquisition time with the disadvantage of accompanying gaze instability and oscillopsia.

### Deficits in Segmental Coordination

Because the rapid, accurate redirection of gaze to new targets during standing requires the coordination of many body parts, it is conceivable that the movement of the most distal part of such linked systems (in this case, the eye) must take into account the progress made by the other segments or subparts (Anastasopoulos et al. 2015; Daye et al. 2014). Single-step gaze shifts at such large eccentricities are only possible if the duration of the time interval available for “saccadic mode” is appropriately prolonged and the switch to gaze “stabilization mode” does not occur until the line of sight has approached the target. According to a conceptual scheme we developed in previous studies with normal subjects (Anastasopoulos et al. 2015), the saccadic mode time interval (understood to be due to inhibition of omnipause brain stem neurons; their reactivation terminates saccadic activity) is not rigidly preprogrammed; it is, instead, thought to be modifiable by global goal feedback and a number of additional signals (such as those of head velocity/eye position; Paré and Guitton 1998). Accordingly, the emergence of oppositely directed slow phases (VOR) and the termination of gaze progression are continually controlled by a mechanism taking into account head-in-space motion. Gaze transfer termination (and VOR emergence) then occurs when the sum of eye-in-orbit and head-in-space displacement has approximately equaled target eccentricity. Once BVL patients decide to foveate the target and mobilize head-trunk segments at a fast speed, they reactivate the gaze stabilization mode (slow phases) prematurely, long before target acquisition, thus generating “non-pause composite” gaze transfers. It is possible that the latter is an adaptive process actively triggered by the loss of vestibular input to stop the hypermetric gaze transfers observed in the acute phase in bi-labyrinthectomized monkeys (Dichgans et al. 1973). Thus our data suggest that head-in-space velocity information is critical for the timely termination of saccadic mode near the target. Because avestibular patients have been shown to be able to execute uninterrupted gaze displacements up to 120° when sitting (Maurer et al. 1998; Sağlam et al. 2014), it can be speculated that, under such experimental conditions, they can make use of head-on-trunk proprioceptive information to accomplish the task. It should not be forgotten, however, that the critical difference between the two protocols is that during sitting, cervical input can theoretically signal head velocity in space fairly accurately, whereas this cannot be the case during trunk-free movements during standing. It can be further concluded that BVL patients are unable to incorporate proprioceptive information from lower body segments (Becker et al. 2002), substituting in this way vestibular and cervical input into the feedback control mechanism governing gaze transfer and gaze termination. Also, feedforward control does not suffice, under these circumstances, as a substitute for the loss of vestibular input (Sağlam et al. 2014).

We found vestibular information to be important not only for the timely termination of gaze saccades but also for shaping online head-in-space and trunk movement, as suggested by the significantly higher jerk cost values in patients, resulting in higher signal-dependent noise (Todorov 2004). The increased trunk sway path after turning implies poor control of trunk movements, the heaviest segment in the body, imposing the risk of losing balance with catastrophic consequences (Liu and Lockhart 2014; Paquette et al. 2016; Schniepp et al. 2017). As such, it should perhaps be unsurprising that trunk movements are also under vestibular control (Day and Reynolds 2005). Thus, although trunk ataxia is a well-recognized feature of central vestibular or cerebellar disease (Carmona et al. 2016; Ogawa et al. 2015), in this study we show that bilateral peripheral vestibular lesions also cause trunk ataxia even in our well-compensated patients.

### Conclusions

Our findings show that online vestibularly derived signals are functionally essential when targets are being acquired at large eccentricities and provide evidence supporting the view of gaze feedback control. The disability imposed to bilateral labyrinthine patients includes, among others, significant delays to target acquisition and trunk ataxia, findings that go well beyond the traditional role of the vestibular system in mediating gaze stability (“compensatory” function of the VOR) and postural control. Most vestibular physiotherapy protocols are heavily biased toward rehabilitation of the compensatory function of the VOR, somewhat neglecting the consistent reports of patients that large gaze transfers (e.g., such as those required before crossing a road) are particularly difficult (anticompensatory function of the VOR). Therapists should incorporate large gaze shifts during standing in their rehabilitation protocols. At least initially, patients should concentrate on large head-eye gaze shifts before rotating the trunk by modifying the existing head-trunk kinematic synergy (Anastasopoulos et al. 2015). In this case patients should be able to rely on cervical input to get head-in-space motion feedback. This would be theoretically feasible, given that both patients and controls chose to move the head-on-trunk far below the limit of 60°–70° (the so-called neutral zone, stiffness remains low up to this limit; McClure et al. 1998).

## GRANTS

Supported from Medical Research Council Grant MR/J004685/1 and the National Institute for Health Research Imperial Biomedical Research Centre is gratefully acknowledged.

## DISCLOSURES

No conflicts of interest, financial or otherwise, are declared by the authors.

## AUTHOR CONTRIBUTIONS

D.A. and A.M.B. conceived and designed research; D.A. and N.Z. performed experiments; D.A. and N.Z. analyzed data; D.A. and A.M.B. interpreted results of experiments; D.A. prepared figures; D.A., N.Z., and A.M.B. drafted manuscript; D.A., N.Z., and A.M.B. edited and revised manuscript; D.A. and A.M.B. approved final version of manuscript.
